# A prospective randomised phase III trial of adjuvant chemotherapy with 5-fluorouracil and leucovorin in patients with stage II colon cancer

**DOI:** 10.1038/sj.bjc.6604011

**Published:** 2007-09-25

**Authors:** W Schippinger, H Samonigg, R Schaberl-Moser, R Greil, R Thödtmann, J Tschmelitsch, M Jagoditsch, G G Steger, R Jakesz, F Herbst, F Hofbauer, H Rabl, P Wohlmuth, M Gnant, J Thaler

**Affiliations:** 1 Division of Oncology, Department of Internal Medicine, Medical University of Graz, A-8036 Graz, Austria; 2 Third Medical Department, Private Medical Paracelsus University Salzburg, A-5020 Salzburg, Austria; 3 St Veit Hospital, Department of Surgery, A-9300 St Veit/Glan, Austria; 4 Division of Oncology, First Medical Department, Medical University of Vienna, A-1090 Vienna, Austria; 5 Department of Surgery, Medical University of Vienna, A-1090 Vienna, Austria; 6 Department of Surgery, Oberpullendorf Hospital, A-7350 Oberpullendorf, Austria; 7 Department of Surgery, Leoben Hospital, A-8700 Leoben, Austria; 8 Statistics Unit, Austrian Breast and Colorectal Cancer Study Group, A-1090 Vienna, Austria; 9 Fourth Medical Department, Wels Hospital, A-4600 Wels, Austria

**Keywords:** adjuvant chemotherapy, colon cancer, stage II

## Abstract

The purpose of this trial was to investigate the efficacy of adjuvant chemotherapy with 5-fluorouracil (5-FU) and leucovorin (LV) in stage II colon cancer. Patients with stage II colon cancer were randomised to either adjuvant chemotherapy with 5-FU/LV (100 mg m^−2^ LV+450 mg m^−2^ 5-FU weekly, weeks 1–6, in 8 weeks cycles × 7) or surveillance only. Five hundred patients were evaluable for analyses. After a median follow-up of 95.6 months, 55 of 252 patients (21.8%) have died in the 5-FU/LV arm and 58 of 248 patients (23.4%) in the surveillance arm. There was no statistically significant difference in overall survival (OS) between the two treatment arms (hazard ratios, HR 0.88, 95% CI 0.61–1.27, *P*=0.49). The relative risk for tumour relapse was higher for patients on the surveillance arm than for those on the 5-FU/LV arm; however, this difference was not statistically significant (HR 0.69, 95% CI 0.45–1.06, *P*=0.09). Consequently, disease-free survival (DFS) was not significantly different between the two trial arms. In conclusion, results of this trial demonstrate a trend to a lower risk for relapse in patients treated with adjuvant 5-FU/LV for stage II colon cancer. However, in this study with limited power to detect small differences between the study arms, adjuvant chemotherapy failed to significantly improve DFS and OS.

Colorectal cancer is the leading cause of cancer-associated deaths in the Western world. Up to 50% of patients with colorectal cancer who undergo potentially curative surgical treatment develop metastatic disease leading to incurability and death (Parkin *et al*, 2001; Jemal *et al*, 2003). The most important prognostic factors influencing the survival of patients with colon cancer are tumour stage determined by the extent of tumour invasion into the bowel wall and involvement of regional lymph nodes. According to the International Union Against Cancer (UICC), stage II colon cancer is defined by tumour penetration through the bowel wall with invasion into the subserosa or deeper, but no regional lymph node involvement nor distant metastases. The overall survival (OS) rate of patients with stage II colon cancer after curative surgery alone is approximately 70–80% (Nauta *et al*, 1989). In patients with stage III colon cancer, as defined by the presence of regional lymph node metastases, several trials have shown adjuvant chemotherapy to improve survival (Moertel *et al*, 1990, 1995; Andre *et al*, 2004). The benefit of adjuvant chemotherapy for patients with stage II disease is still a matter of controversy. Results of a single randomised trial failed to demonstrate an improvement of OS by adjuvant chemotherapy in patients with stage II colon cancer (Moertel *et al*, 1995). The results of pooled analyses of data from four National Surgical Adjuvant Breast and Bowel Project (NSABP) adjuvant studies and of the International Multicentre Pooled Analysis of B2 Colon Cancer (IMPACT B2), including several trials with stage II colon cancer patients, demonstrated controversial results with regard to the survival benefit achieved by adjuvant chemotherapy (IMPACT B2 Investigators, 1999; Mamounas *et al*, 1999).

The purpose of the trial reported here, initiated and conducted by the Austrian Breast and Colorectal Cancer Study Group (ABCSG), was to examine the impact of adjuvant chemotherapy with 5-fluorouracil (5-FU) and leucovorin (LV) on OS and disease-free survival (DFS) in patients following curative surgery for stage II colon cancer.

## MATERIALS AND METHODS

This multicentre phase III trial involved 31 participating hospitals. The protocol was approved by the ethics committees at the participating institutions. Enrolment of patients began in November 1993 and was finished in June 2003.

### Patient selection

To be eligible for this study, patients had to fulfil the following inclusion criteria: written informed consent to participate in this trial, histologically proven stage II colon carcinoma according to the UICC (T3-T4, N0, M0), potentially curative resection without gross or microscopic evidence of residual disease, age between 18 and 80 years, World Health Organization (WHO) performance status of 0 or 1, absence of severe concomitant disease and other malignancies, and adequate bone marrow, renal and hepatic function. The following were applied as exclusion criteria: prior or concomitant chemotherapy, immunotherapy or radiotherapy, and carcinoma of the rectum defined as tumour below the anatomical rectosigmoidal borderline or within 16 cm from the anal verge measured by a non-flexible rectoscope. Other exclusion criteria included the presence of metastasis or an interval exceeding 42 days between surgery and the start of adjuvant therapy.

Written informed consent was obtained from all patients participating in this trial.

Monitoring visits were performed regularly at the participating centres to inspect the original data concerning eligibility and to review documented chemotherapy and follow-up data.

### Surgical treatment

A tumour of the caecum, the ascending colon, or the hepatic flexure was resected with a right hemicolectomy, resecting the right colic artery and the ileocolic artery. A tumour of the colon transversum was treated by resection of the appropriate part of the colon including the hepatic and splenic flexure. Carcinomas of the descending colon were resected by a left hemicolectomy including the left colic artery. Finally, sigmoid tumours were treated by a sigmoid resection including the inferior mesenteric blood vessels. The protocol required precise removal of locoregional lymph nodes.

### Randomisation and stratification procedures

Treatment allocation was obtained by contacting the central randomisation office by telephone. Randomisation was performed by computer assigning the patients to one of two post-operative treatment arms: (1) 5-FU/LV, (2) surveillance only. The method of Pocock and Simon (1975) was employed to marginally balance the following criteria: gender, age (⩽65 *vs* >65–80 years), tumour size (T3 *vs* T4), tumour differentiation (G1/2 *vs* G3/4), and centre.

### Chemotherapy

In the chemotherapy arm (study arm 1), patients received adjuvant therapy consisting of LV 100 mg m^−2^ administered as an intravenous bolus, followed by 5-FU 450 mg m^−2^ as an intravenous short infusion over 15 min given once weekly for 6 weeks in each 8-week cycle for a total of seven cycles (=56 weeks of therapy). As per protocol, adjuvant chemotherapy was required to be started within 42 days after tumour resection. In the control arm (study arm 2), patients were assigned to surveillance only.

In study arm 1, dose modifications were required for haematological or other severe toxicity. According to the protocol, the 5-FU dosage was reduced to 350 mg m^−2^ for subsequent therapy cycles when patients developed grade II toxicity according to the WHO criteria. In case of occurrence of WHO grade III toxicity, chemotherapy was delayed for 1 week and continued with 5-FU at a dosage of 350 mg m^−2^. Chemotherapy was discontinued in any case of WHO grade IV toxicity, or when grade III toxicity re-occurred in spite of previous 5-FU dose reduction. In case of leucopenia and/or thrombocytopenia, continuation of chemotherapy was delayed until a whole leucocyte count of ⩾3.0 × 10^9^ l^−1^ and/or a platelet count of ⩾100 × 10^9^ l^−1^ was reached.

### Follow-up

Patients were evaluated every 3 months during the first year post-randomisation (=treatment period in arm 1), every 6 months during years 2–5, and once yearly until year 10 after randomisation. A physical examination with evaluation of body weight and performance status, determination of blood count, serum liver function parameters, carcinoembryonic antigen and tests for occult blood in stool, as well as chest radiography and ultrasonography of the liver were performed at each follow-up visit. Colonoscopy was required every 6 months during the first 6 years after randomisation, then once a year.

### Statistical methods

The trial was designed to detect a difference in OS of 10% between the two study arms (70 *vs* 80%) with a power of 85% and a two-sided significance level of 0.05, so that approximately 318 patients would be recruited to each arm in 3 years. The final analysis was planned 7 years after the study began. After 535 patients had been accrued, recruitment was stopped ahead of schedule for low accrual rates.

All patient data were collected at the Study Group's central data office and processed and analysed applying SAS software (SAS Institute, Cary, NC, USA). Distribution of prognostic factors to the treatment arms was described with frequencies for categorial variables and with medians for continuous data and tested with the *χ*^2^-test and Kruskal–Wallis test, respectively.

Overall survival was defined as the interval between date of randomisation and date of last visit or date of death, independent of the cause of death. Disease-free survival was defined as the interval between randomisation and either recurrence of colon cancer, occurrence of metastases, occurrence of a second primary cancer, or death without evidence of recurrence, or date of last visit.

Cancer-specific survival was defined as the interval between date of randomisation and date of last visit or date of death caused by colon cancer.

Overall survival, DFS, and cancer-specific survival were estimated and graphically presented according to the method of Kaplan and Meier (1958). Differences between curves were assessed by the Mantel log-rank test for censored survival data (Mantel, 1966). The Cox proportional hazards model (Cox, 1972) was used to assess the prognostic values of treatment, gender, age, tumour grading, tumour stage, tumour localisation, and number of surgically removed lymph nodes in univariate and multivariate analyses, quantifying their effect by hazard ratios (HR). Backward, forward, and stepwise procedures were used and compared to identify the final model with a threshold of 10% for the variables to stay in or enter the model. The results were solid and the results were taken from the stepwise procedure. Corresponding 95% confidence intervals (CI) are also given. All *P*-values given are two sided.

## RESULTS

### Patient characteristics

A total of 535 patients were included in this prospective randomised trial. Thirty-three randomised patients were found to be ineligible and were therefore not included in the analysis. Ineligibility of patients was determined by the central data monitoring committee of this trial. The reasons for ineligibility of patients in the two study arms are shown in Table 1. Two patients were not evaluable for analysis for lack of follow-up data. Statistical analyses were performed with the data of the remaining 500 eligible and evaluable patients according to the intention-to-treat principle. Baseline patient and tumour characteristics are reported in Table 2. The patient and tumour characteristics were well balanced between the two study arms. The median follow-up time for the study population was 95.6 months (range 0–148.9 months).

The median number of lymph nodes resected was 17 (range 5–68).

### Treatment adherence

Adjuvant chemotherapy with seven cycles of 5-FU/LV as planned in the protocol was administered to 164 of 252 (65.1%) evaluable patients on the chemotherapy arm. Seven (2.8%) patients on study arm 1 received no chemotherapy, 15 (6.0%) patients received one cycle, 14 (5.6%) patients two cycles, 12 (4.8%) patients three cycles, 14 (5.6%) patients four cycles, 8 (3.2%) patients five cycles, and 18 (7.1%) patients six cycles.

In most of the cases (72.3%), the reason for treatment discontinuation was the patients’ wish to stop therapy.

Toxicity-induced dose reduction of 5-FU was performed according to the protocol in 44 (17.5%) patients.

### Tumour relapse

To date, 83 of the 500 patients have relapsed. Tumour relapse, as defined by occurrence of metastatic disease or local recurrence, was documented in 35 of 252 patients (13.9%) on chemotherapy and in 48 of 248 patients (19.4%) in the control group. In univariate analysis, the relative risk of relapse in the patient group treated with adjuvant chemotherapy (study arm 1) was lower than in the patient group that had received no adjuvant chemotherapy (study arm 2); however, the difference was statistically not significant (HR 0.69, 95% CI 0.45–1.06, χ^2^-test: *P*=0.09).

**Figure 1:**
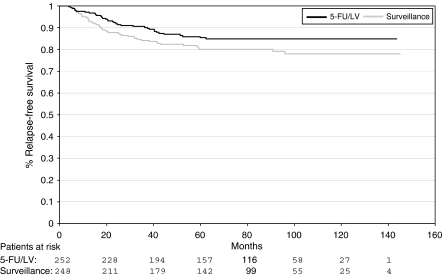
Relapse-free survival: adjuvant chemotherapy with 5-fluorouracil (5-FU)/leucovorin (LV) *vs* surveillance.

Kaplan–Meier curves for relapse-free survival are shown in Figure 1.

### Disease-free survival

Disease-free survival was statistically not significantly different between patients on the surveillance arm and those treated with adjuvant 5-FU/LV (76 events in the surveillance arm, 75 events in the chemotherapy arm; HR 0.95, 95% CI 0.69–1.31, χ^2^-test: *P*=0.77).

### Overall survival

Up to now, 113 of 500 evaluable patients have died: 55 of 252 patients (21.8%) on the chemotherapy arm (study arm 1) and 58 of 248 patients (23.4%) in the surveillance group (study arm 2). There was no statistically significant difference in OS between the two treatment arms (HR 0.88, 95% CI 0.61–1.27, χ^2^-test: *P*=0.49).

**Figure 2:**
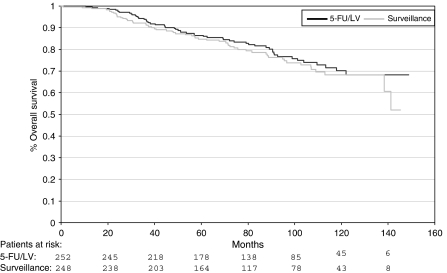
Overall survival: adjuvant chemotherapy with 5-fluorouracil (5-FU)/leucovorin (LV) *vs* surveillance.

Kaplan–Meier curves for OS are presented in Figure 2.

### Cancer-specific survival

In 71 patients of the entire study population, death was related to colon cancer.

Colon cancer-related death was documented in 33 patients (13.1%) on the chemotherapy arm (study arm 1) and in 38 patients (15.3%) on the control arm (study arm 2). The relative risk for colon cancer-related death was not statistically significantly different between the two study arms (HR 0.83, 95% CI 0.52–1.33, χ^2^-test: *P*=0.44).

**Figure 3:**
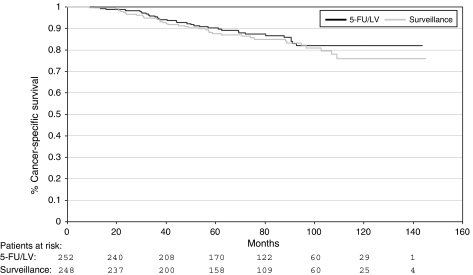
Cancer-specific survival: adjuvant chemotherapy with 5-fluorouracil (5-FU)/leucovorin (LV) *vs* surveillance.

Kaplan–Meier curves for cancer-specific survival are shown in Figure 3.

Additional analyses on a total of 513 patients, also including 13 randomised patients who were not eligible, but who had signed and did not withdraw informed consent, did not change the results. There was no statistically significant difference between the two study arms in relapse-free survival (HR 0.68, 95% CI 0.44–1.05, *P*=0.08), DFS (HR 0.96, 95% CI 0.70–1.32, *P*=0.81), OS (HR 0.89, 95% CI 0.62–1.29, *P*=0.55), or cancer-specific survival (HR 0.82, 95% CI 0.51–1.30, *P*=0.39).

### Multivariate analyses of prognostic factors

Cox regression analysis evaluating prognostic parameters for tumour relapse, as defined by occurrence of metastatic disease or local recurrence, demonstrated the number of surgically removed lymph nodes as statistically significant prognostic factor for tumour relapse. The risk for tumour relapse was significantly lower in patients with more than 12 lymph nodes removed. The impact of tumour localisation at caecum and right colon on the risk of tumour relapse was of borderline significance (*P*=0.06). Patients with tumours located in caecum and right colon had a risk of tumour relapse that was 1.55 times that of patients with tumours in the left colon and sigmoid or in flexures and transverse colon. Age, gender, tumour size, grading, and tumour localisation at flexures and transverse colon had no significant impact on the risk of tumour relapse.

Cox regression analysis examining prognostic factors for DFS revealed age and again the number of surgically removed lymph nodes, and tumour localisation at caecum and right colon as significant prognostic factors. The risk for a DFS-relevant event increased by 4% with each additional year of patients’ age at the time of randomisation. Gender, grading, tumour size, and tumour localisation at flexures and transverse colon did not significantly influence DFS in the multivariate analysis.

With regard to OS, age, the number of surgically removed lymph nodes, and again tumour localisation at caecum and right colon were found to be significant prognostic variables. Gender, tumour size, grading, and tumour localisation at flexures and transverse colon demonstrated no significant impact on OS in the Cox model.

Cox analysis of prognostic factors for cancer-specific survival again revealed the number of surgically removed lymph nodes and tumour localisation at caecum and right colon as the only statistically significant parameters.

The results of multivariate analyses of prognostic factors for tumour relapse, DFS, OS, and cancer-specific survival are shown in Tables 3, 4, 5 and 6.

### Toxicity

In 4.5% of the patients, grade 3 and grade 4 toxicities were documented during adjuvant chemotherapy with 5-FU/LV. The distribution of grade 3 and grade 4 toxic effects is shown in Table 7.

## DISCUSSION

The results of the present trial comparing adjuvant chemotherapy with 5-FU/LV to surveillance after curative surgery for stage II colon cancer demonstrated a non-significant reduction of the relative risk for tumour relapse in patients on the chemotherapy arm. The results of our trial did not demonstrate a significant impact of adjuvant chemotherapy with 5-FU/LV on DFS and OS in patients with stage II colon cancer. Previous studies investigating a possible survival benefit gained by adjuvant chemotherapy in patients with stage II colon cancer have resulted in controversial findings. Inter-group trial INT-0035 randomised patients with stage II and stage III colon cancer to either 5-FU/levamisole or surgery alone. The trial revealed a significant survival benefit in patients with stage III colon cancer treated with adjuvant chemotherapy, but no statistically significant survival difference between the chemotherapy and surgery-alone arm within the small group of patients with stage II disease (Moertel *et al*, 1990, 1995).

Between 1977 and 1990, the NSABP conducted four adjuvant chemotherapy trials in patients with stage II and III colon cancer. These trials compared different adjuvant chemotherapy regimens with one another or with surgery alone (Wolmark *et al*, 1988, 1990, 1993, 1999). A pooled analysis comparing the best treatment arm with the inferior arm of each of these trials revealed a 30% reduction in overall mortality and an absolute survival benefit of 5% for patients with stage II disease, regardless of presence or absence of high-risk parameters, such as bowel obstruction, perforation, or extension to adjacent organs (Mamounas *et al*, 1999).

Whereas the results of the NSABP pooled analysis clearly suggested to recommend adjuvant chemotherapy to patients with stage II colon cancer, the data of another pooled analysis, conducted by the IMPACT B2 investigators, did not support the routine use of adjuvant chemotherapy in patients with stage II disease. The IMPACT B2 analysis included 1016 patients with stage II colon cancer in five separate trials who were randomised to adjuvant chemotherapy with 5-FU/LV or observation after curative surgery. After a median follow-up duration of 5.75 years, no statistically significant difference in event-free survival or OS was observed between the two treatment categories (IMPACT B2 Investigators, 1999).

The American Society of Clinical Oncology (ASCO) published recommendations on adjuvant chemotherapy for stage II colon cancer in 2004 (Benson *et al*, 2004). These recommendations were guided by a literature-based meta-analysis including 37 trials and 11 meta-analyses, altogether demonstrating a small improvement of DFS by 5–10%, but no statistically significant improvement of OS by adjuvant chemotherapy (Figueredo *et al*, 2004). The authors of the ASCO recommendations thus concluded that direct evidence from randomised controlled trials does not support the routine use of adjuvant chemotherapy in patients with stage II colon cancer.

Results of the Quick and Simple and Reliable (QUASAR) 1 trial, investigating the impact of adjuvant chemotherapy on survival in 3238 patients with colorectal cancer (91% stage II), demonstrated for patients with stage II disease a small, but statistically significant absolute 5-year survival benefit of 2.9% on the chemotherapy arm after a median follow-up of 4.2 years. The authors of this presently largest single trial in stage II colon cancer concluded that ‘chemotherapy produces a small (1–5%) survival benefit for stage II patients, sufficient to outweigh the inconvenience and cost for high-risk and younger patients’ (Gray *et al*, 2004).

The Multicenter International Study of Oxaliplatin/5-FU/ Leucovorin in the Adjuvant Treatment of Colon Cancer (MOSAIC) compared the efficacy of adjuvant 5-FU/LV/oxaliplatin to 5-FU/LV in patients with stage II and III colon cancer. Results of the MOSAIC trial demonstrated a statistically significant improvement of DFS in the 5-FU/LV/oxaliplatin arm in both the subgroups of patients with stage II and those with stage III disease (Andre *et al*, 2004).

These data demonstrate that adjuvant chemotherapy in stage II colon cancer is effective, though the effect on survival is small. In our trial, adjuvant chemotherapy decreased the relative risk for tumour relapse, yet with no significant impact on DFS or OS. This trial was designed to detect an absolute difference in OS of 10% between the two study arms. In addition, the planned patient number was not reached, a result being that the trial is not sufficiently powered to detect the small survival difference as observed in other trials. Due to the fact that the target number of patients recruited was not reached, the power to detect a difference in OS of 10% was reduced from 85 to 70.5%. The power of this trial to detect an absolute difference in 5-year survival of 2.9% as observed in the QUASAR 1 trial is 16.6%.

Multivariate analysis in the present trial showed the number of lymph nodes resected to be a significant prognostic factor for the risk of tumour relapse, DFS, OS, and cancer-specific survival. Patients with more than 12 nodes analysed exhibited a significantly better prognosis compared to patients in whom 12 or less lymph nodes had been examined. This association between the number of lymph nodes resected and prognosis of patients with node-negative colon cancer confirms the results emanating from previous studies (Le Voyer *et al*, 2003; Chen and Bilchik, 2006). A possible explanation for this finding is that patients with fewer nodes analysed might be understaged and at higher risk for residual tumour disease after surgery.

In conclusion, the results of this prospective randomised trial with limited power to detect relevant differences between the study arms failed to demonstrate a statistically significant impact of adjuvant chemotherapy with 5-FU/LV on DFS and OS in patients following curative surgery for stage II colon cancer. Thus, the findings of this trial do not support the general use of adjuvant chemotherapy in all patients with stage II colon cancer.

## Figures and Tables

**Table 1:** Reasons for ineligibility of patients in the two study arms

	**5-FU/LV**	**Surveillance**
	***n***	***n***
*Violation of inclusion criteria*		
Missing or withdrawn informed consent	8	14
Inadequate removal of locoregional lymph nodes	0	1
Metastases in locoregional lymph nodes	5	2
Missing examinations excluding metastatic disease	2	0
Severe concomitant disease	0	1

Total number of patients ineligible	17	18

5-FU=5-fluorouracil; LV=leucovorin.

**Table 2:** Patient and tumour characteristics

	**5-FU/LV**	**Surveillance**	
	***n* (%)**	***n* (%)**	***P*-value**
Number of patients	252 (50.4)	248 (49.6)	
Median age (years)	64.5	65.4	0.60
Age range	28.5–79.3	30.0–79.7	
Male/female	137/115 (54.4/45.6)	134/114 (54.0/46.0)	0.94

*T category*			0.95
T3	217 (86.1)	214 (86.3)	
T4	35 (13.9)	34 (13.7)	

*Grading*			0.71
G1 and G2	204 (81.0)	204 (82.3)	
G3 and G4	48 (19.0)	44 (17.7)	

*Tumour localisation*			0.85
Caecum and right colon	76 (30.2)	69 (27.8)	
Left colon and sigmoid	122 (48.4)	124 (50.0)	
Flexures and transverse colon	54 (21.4)	55 (22.2)	
Median number of resected lymph nodes	17	17	0.82

5-FU=5-fluorouracil; LV=leucovorin.

**Table 3:** Multivariate analysis: significant prognostic factors for tumour relapse

	**HR**	**95 % CI**	***P*-value**
Number of resected lymph nodes	0.44	0.28–0.68	<0.01
Tumour located in caecum and right colon	1.55	0.98–2.44	0.06

HR=hazard ratios; CI=confidence interval.

For the HR the reference category for categorical covariates was caecum and right colon for localisation of tumour, >12 lymph nodes for number of resected lymph nodes.

**Table 4:** Multivariate analysis: significant prognostic factors for DFS

	**HR**	**95 % CI**	***P*-value**
Age	1.04	1.02–1.05	<0.01
Number of resected lymph nodes	0.59	0.42–0.82	<0.01
Tumour located in caecum and right colon	1.64	1.17–2.28	<0.01

HR=hazard ratios; CI=confidence interval.

For the HR the reference category for categorical covariates was caecum and right colon for localisation of tumour, >12 lymph nodes for number of resected lymph nodes.

**Table 5:** Multivariate analysis: significant prognostic factors for OS

	**HR**	**95 % CI**	***P*-value**
Age	1.05	1.05–1.07	<0.01
Number of resected lymph nodes	0.49	0.34–0.72	<0.01
Tumour located in caecum and right colon	1.81	1.24–2.66	<0.01

HR=hazard ratios; CI=confidence interval.

For the HR the reference category for categorical covariates was caecum and right colon for localisation of tumour, >12 lymph nodes for number of resected lymph nodes.

**Table 6:** Multivariate analysis: significant prognostic factors for cancer-specific survival

	**HR**	**95 % CI**	***P*-value**
Number of resected lymph nodes	0.54	0.33–0.88	0.01
Tumour located in caecum and right colon	1.68	1.03–2.74	0.04

HR=hazard ratios; CI=confidence interval.

For the HR the reference category for categorical covariates was caecum and right colon for localisation of tumour, >12 lymph nodes for number of resected lymph nodes.

**Table 7:** Grade 3 and 4 toxicities of 245 patients with toxicity data available on study arm 1

	***n***	**%**
Nausea	4	1.6
Diarrhoea	6	2.5
Stomatitis	0	0
Fever	0	0
Leucopenia	0	0
Thrombopenia	1	0.4
Alopecia	0	0

## Appendix

Apart from the authors of this article, members of the Austrian Breast and Colorectal Cancer Study Group participating in this trial included the following:

H Bacher, R Bartsch, T Bauernhofer, A Berger, J Berger, M Bergmann, C Bittermann, O Böckl, K Czerwenka, D Depisch, W Draxler, P Dubsky, A El-Shabrawi, J Freisinger, KM Freudenthaler, K Haider, M Hanak, B Hartmann, H Hauser, J Hebenstreit, E Hell, G Hofmann, D Hussian, G Jatzko, C Kainz, D Kandioler, A Kasparek, C Kiendler, R Kolb, P Konstantinuik, G Kosina, A Kretschmer, P Krippl, S Kriwanek, L Kronberger, I Kuehrer, I Kuss, W Kwasny, M Lang, N Langer, U Langsenlehner, A Lenauer, G Locker, G Loncsar, H Ludwig, I Luisser, H Luschnik, K Mach, P Mayer, G Michlmayr, HJ Mischinger, M Mittlböck, E Moritz, A Obermair, E Nessler, T Niernberger, D Nitsche, D Öfner, E Ostermann, I Papadi, C Papp, A Pertl, J Pfeifer, F Ploner, U Pluschnig, R Pointner, M Prochaska, R Pruscha, R Punzengruber, C Rass, A Reiner, G Reiner, S Reinisch, M Riegler, S Roka, G Rosanelli, M Rudas, G Russ, P Sandbichler, R Schandalik, M Schemper, M Schmid, W Schwaiger, U Sevelda, H Spoula, T Stein, P Steindorfer, A Stift, H Stöger, C Tausch, B Teleky, K Tögel, G Tschurtschenthaler, M Umlauft, N Vavra, P Wagner, G Wahl, M Wehrschütz, W Weitzer, C Wenzel, B Zeh, R Zwrtek, and C Zielinski.
